# Development and Psychometric Assessment of a Measurement Instrument for Functioning and Function‐Affecting Factors in Women With Breast Cancer: A Rasch Analysis Approach

**DOI:** 10.1111/jep.70705

**Published:** 2026-10-28

**Authors:** Farkhondeh Jamshidihosseinabadi, Maryam Farzad, Enayatollah Bakhshi, Seyed Ali Hosseini, Amir Hossein Emami, Kamran Roudini, Mehrzad Mirzania, Farhad Shahi, Mohsen Esfandbod, Mojtaba Habibi Asgarabad

**Affiliations:** ^1^ Department of Occupational Therapy School of Allied Medical Sciences, Zanjan University of Medical Sciences Zanjan Iran; ^2^ School of Physical Therapy, Department of Health and Rehabilitation Sciences University of Western Ontario. Roth McFarlane Hand and Upper Limb Center, St. Joseph's Hospital, London, Ontario, Canada, Department of Occupational therapy, University of Social Welfare and Rehabilitation Sciences Tehran Iran; ^3^ Department of Occupational Therapy University of Social Welfare and Rehabilitation Sciences Tehran Iran; ^4^ Department of Biostatistics University of Social Welfare and Rehabilitation Sciences Tehran Iran; ^5^ Department of Hematology Oncology Imam Khomeini Hospital, Tehran University of Medical Sciences Tehran Iran; ^6^ Department of Internal Medicine School of Medicine, Imam Khomeini Hospital, Tehran University of Medical Sciences Tehran Iran; ^7^ Department of Hematology and Medical Oncology Cancer Research Center, Cancer Institute of Iran, Imam Khomeini Hospital Complex, Tehran University of Medical Sciences Tehran Iran; ^8^ Department of Medical Oncology Cancer Research Center, Cancer Institute, Imam Khomeini Hospital Complex, Tehran University of Medical Sciences Tehran Iran; ^9^ Department of Hematology and Oncology Imam Khomeini Hospital Complex, Tehran University of Medical Sciences Tehran Iran; ^10^ Health Promotion Research Center Iran University of Medical Sciences Tehran Iran; ^11^ Department of Psychology Norwegian University of Science and Technology Trondheim Norway; ^12^ Department of Psychology Faculty of Humanities and Social Sciences, Istinye University Istanbul Türkiye

**Keywords:** breast cancer, functioning, ICF core set, psychometric properties, Rasch analysis, structural validity

## Abstract

**Purpose:**

To develop and psychometrically evaluate an instrument for assessing functioning and function‐affecting factors in women with breast cancer, based on the International Classification of Functioning, Disability and Health (ICF) Core Set for Breast Cancer.

**Methods:**

This methodological study combined a systematic literature review, qualitative interviews with women with breast cancer, and expert consultation to develop the questionnaire. Face validity was assessed in 10 women with breast cancer, content validity by 10 experts, and structural validity and reliability in 305 women with breast cancer. Rasch analysis was performed using dichotomized item responses, and confirmatory factor analysis (CFA) was conducted in R using the WLSMV estimator for ordered categorical variables. Internal consistency was evaluated using McDonald's Omega.

**Results:**

Following content validation, the preliminary questionnaire contained 70 items (40 functioning and 30 function‐affecting factors). Rasch analysis identified misfitting items and resulted in a final 55‐item instrument comprising 32 functioning items and 23 function‐affecting items. CFA supported the proposed multidimensional structure after theoretically justified model refinement. The functioning section demonstrated satisfactory model fit (CFI = 0.976, TLI = 0.974, RMSEA = 0.040, 90% CI = 0.034–0.046), while the function‐affecting factors section also demonstrated acceptable fit (CFI = 0.979, TLI = 0.977, RMSEA = 0.054, 90% CI = 0.046–0.062). Although several items showed relatively low standardized factor loadings, they were retained because of acceptable Rasch fit, satisfactory content validity, and conceptual importance. McDonald's Omega indicated acceptable internal consistency across the retained subscales.

**Conclusion:**

The findings provide preliminary evidence supporting the reliability and structural validity of this ICF‐based instrument for assessing functioning and function‐affecting factors in women with breast cancer. Further validation in independent populations is recommended before broader clinical implementation.

## 1 Introduction

Breast cancer (BC) is the most prevalent malignancy among women worldwide. According to the American Cancer Society, nearly 300,000 new BC cases were reported in the United States in 2022, with approximately one‐sixth of these women succumbing to the disease [1]. In Iran, the prevalence of cancer is also on the rise, with women being diagnosed with BC nearly a decade earlier than their Western counterparts [2, 3]. Advances in BC treatment have improved survival rates to over 50% through surgery, chemotherapy, and radiation therapy [4]. Nevertheless, many survivors experience long‐term psychosocial and physical complications that profoundly affect their daily lives [5]. These complications include disturbances in body image and self‐concept [6, 7], social isolation, difficulties in family and sexual relationships, and fear of recurrence and death [8, 9]. Furthermore, patients often face physical limitations such as pain, reduced range of motion, and lymphedema [10, 11, 12, 13]. These limitations interfere with their ability to engage in domestic tasks, return to work, and maintain social roles, which in turn influence their economic status, interpersonal relationships, psychological well‐being, and overall quality of life [14].

Given these challenges, rehabilitation medicine has increasingly sought to integrate functional assessment into cancer care [15]. However, there is currently no systematic framework that comprehensively captures the full spectrum of BC‐related functioning and disability [16]. The International Classification of Functioning, Disability, and Health (ICF) provides a globally recognized biopsychosocial model for describing health conditions [17, 18, 19]. The ICF core set for BC encompasses body functions, body structures, activities and participation, and environmental factors [17, 18, 19, 20], thereby offering a systematic framework to document and assess functioning in this population. Despite its utility, functional assessment in BC survivors has traditionally relied on generic or condition‐specific Patient‐Reported Outcome Measures (PROMs) [21, 22, 23]. A systematic review using the COSMIN checklist found that existing instruments only partially covered the dimensions of functioning and provided limited evidence of psychometric robustness [24]. Moreover, most of these tools primarily emphasize activity and physical functioning, while underrepresenting personal and environmental factors—domains that substantially influence recovery and quality of life [24, 25, 26].

Previous studies have developed several functional assessment instruments for women with breast cancer, including generic tools such as the DASH, UEFI, WHODAS 2.0, and PSFS [27, 28, 29, 30, 31, 32], as well as disease‐specific scales like the Lymph‐ICF and BCSQ‐BC [25, 26, 33, 34]. While these instruments have demonstrated varying levels of reliability and validity, they often lack comprehensive coverage of personal, emotional, and environmental factors, or fail to incorporate patient perspectives from different stages of the disease trajectory [25, 35]. Notably, most tools primarily emphasize physical function and activity participation while giving insufficient attention to the psychosocial and contextual dimensions that are crucial for long‐term survivors [26, 35].

Furthermore, although these instruments report acceptable psychometric properties, several shortcomings remain. For example, responsiveness is insufficient in some scales [32, 34], and content validity is often assessed qualitatively rather than quantitatively [25, 26]. Additionally, the integration of ICF categories is partial and does not capture unique domains identified by patients living in the chronic stage of breast cancer [25, 35]. These gaps underline the need for a comprehensive, ICF‐based instrument that integrates both functioning domains and function‐affecting factors to more accurately assess the lived experiences of women with breast cancer.

Recent evidence further suggests that disease‐specific measures demonstrate greater sensitivity to health status changes compared to generic instruments [36]. This highlights a critical gap: no available tool comprehensively captures the multidimensional aspects of functioning in women with breast cancer within the ICF framework.

Therefore, the present study aims to develop and psychometrically evaluate a comprehensive, breast cancer–specific instrument grounded in the ICF core set. This instrument is intended to assess the functioning, roles, and participation of women with breast cancer, while considering personal and environmental determinants that shape their health outcomes.

## 2 Materials and Methods

### 2.1 Study Design

This methodological study was conducted to develop and psychometrically evaluate a measurement instrument designed to assess functioning and function‐affecting factors in women with breast cancer (BC). The design, conduct, and reporting of the study were guided by the COnsensus‐based Standards for the selection of health Measurement INstruments (COSMIN) reporting guideline (version 2.0), which provides methodological recommendations for studies on the development and evaluation of patient‐reported outcome measures [37]. The study followed a sequential exploratory mixed‐methods design consisting of two major phases:

1. Instrument Development
2. Psychometric Evaluation

The development process was grounded in the International Classification of Functioning, Disability, and Health (ICF) comprehensive core set for breast cancer to ensure conceptual and clinical relevance. The instrument was developed during the first phase through evidence synthesis and qualitative exploration, followed by quantitative psychometric testing in the second phase. The overall study workflow and sequential phases of instrument development and validation are illustrated in Figure 1.

**Figure 1:**
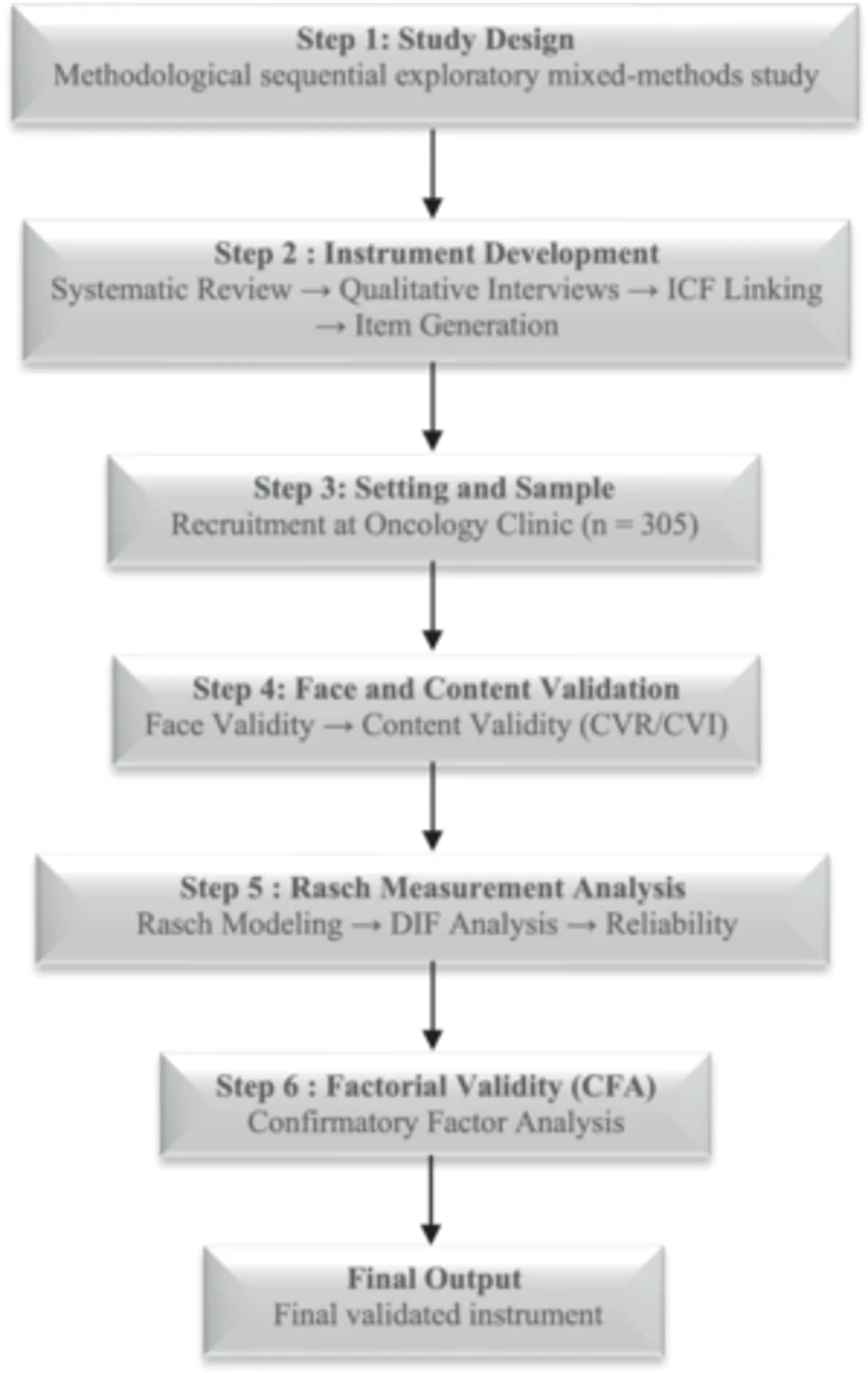
Flowchart of the instrument development and validation process.

### 2.2 Instrument Development Phase

#### 2.2.1 Systematic Review

The review served as the conceptual foundation for identifying domains and existing measurement gaps, which guided the subsequent qualitative exploration. An initial pool of items was derived from a previously conducted systematic review of existing instruments assessing functioning in women with BC [24]. This review identified key domains, measurement gaps, and commonly used constructs relevant to functional outcomes.

#### 2.2.2 Qualitative Study

Semi‐structured interviews were conducted with 16 women diagnosed with breast cancer (BC) and 22 medical and rehabilitation specialists. These interviews aimed to explore functional problems, relevant categories for the tool, and patient experiences. The qualitative data informed item generation, refinement, and alignment with the ICF core set for BC [35].

#### 2.2.3 Item Generation and Refinement

An initial item pool was generated by triangulating findings from the systematic review, qualitative data, and relevant ICF categories. Redundant, ambiguous, or overlapping items were reviewed and refined through iterative discussions within the research team to ensure conceptual clarity and content coverage.

All items were initially scored using an 11‐point numeric rating scale ranging from 0 to 10. The selection of this response format was based on its widespread use in health‐related outcome measurement, particularly for assessing intensity and severity of symptoms and functional limitations. Numeric rating scales (0–10) allow finer discrimination across levels of functioning and are commonly recommended for patient‐reported outcome measures evaluating subjective experiences. The response format was reviewed and approved during expert panel discussions to ensure clarity, interpretability, and consistency with existing clinical assessment practices.

### 2.3 Setting and Sample

#### 2.3.1 Setting

The quantitative phase was conducted at the oncology clinic of Imam Khomeini Hospital, Tehran, Iran.

#### 2.3.2 Participants

Between November 2021 and March 2022, eligible women with a confirmed diagnosis of breast cancer were consecutively recruited from the oncology clinic. Eligible participants were women aged 18 years or older, diagnosed at stages I–III, and who had completed their primary treatments (surgery, radiotherapy, and chemotherapy) at least 6 months prior to enrollment. Hospitalized patients undergoing active treatment were excluded.

To ensure that the instrument captured stable functioning rather than acute treatment‐related effects, only women who had completed active treatment at least 6 months earlier were included. This interval allowed recovery from acute treatment effects and better reflected survivorship‐related functioning, improving the conceptual validity of the measurement.

Demographic and clinical data, including age, marital status, education level, employment, type of surgery, and affected side, were collected. All participants provided written informed consent. Ethical approval was obtained from the Ethics Committee of the University of Social Welfare and Rehabilitation Sciences (IR.USWR.REC.1398.198).

### 2.4 Psychometric Evaluation

#### 2.4.1 Content Validity (Including Face Validity)

Content validity was evaluated using both qualitative and quantitative approaches and encompassed assessment of item relevance, comprehensiveness, and comprehensibility. In line with contemporary measurement theory and COSMIN recommendations, face validity was considered a component of content validity, reflecting the perceived clarity and appropriateness of items from the target population's perspective.

#### 2.4.2 Qualitative Content Validity (Face Validity)

To evaluate comprehensibility and clarity from the patients' perspective, qualitative interviews were conducted with 10 women with breast cancer who met the study criteria. Participants were asked to comment on wording, clarity, readability, and ease of completion. Minor linguistic refinements were made based on their feedback, without removing any items at this stage.

#### 2.4.3 Quantitative Content Validity

Quantitative content validity was assessed using the content validity ratio (CVR) and content validity index (CVI). Fourteen experts in medical and rehabilitation sciences with at least 5 years of experience in breast cancer care were invited to evaluate the items.

For CVR, experts rated items as essential [3], useful but not essential [2], or not necessary [1]. For CVI, they evaluated relevance, simplicity, and clarity on a 4‐point Likert scale (from not relevant to highly relevant) [38].

Qualitative feedback from the same experts was also obtained regarding grammar, terminology, spelling, comprehensibility, and item placement. Based on expert panel feedback, several types of item‐level amendments were undertaken to improve content representativeness and clarity. These amendments included [1]: linguistic revisions to enhance readability and cultural appropriateness [2] conceptual clarification of items whose meanings were considered vague or overlapping [3]; merging of redundant items addressing identical ICF categories [4] splitting of complex items containing more than one concept into separate items to ensure unidimensional measurement; and [5] reclassification of items into more appropriate domains when their conceptual alignment was questioned. All modifications were performed through iterative consensus discussions within the research team and expert panel to preserve the original construct while improving precision. A detailed record of these amendments is presented in Supporting Information S1.

#### 2.4.4 Expert Panel for Quantitative Content Validation

Although patient perspectives are increasingly valued in outcome measure development, quantitative content validation (CVR and CVI) traditionally relies on expert panels to ensure conceptual accuracy and theoretical coherence. This expert‐based approach aligns with standard psychometric procedures [38, 39]. In this study, patients' insights were incorporated during the earlier qualitative phase to capture experiential aspects, whereas expert evaluations were prioritized during quantitative content validation to safeguard clinical relevance. This dual‐phase integration of patient and expert input reflects current best practices in instrument development [40]. Nevertheless, because quantitative content validation relied primarily on expert judgments, it remains possible that patients with different levels of health literacy, cultural backgrounds, or lived experiences may interpret some retained items differently. This issue is acknowledged as a study limitation and warrants further investigation in future studies involving more diverse patient populations.

#### 2.4.5 Structural Validity and Reliability

The Rasch model was employed to examine structural validity and reliability. This model belongs to the family of modern test theory approaches (Item Response Theory, IRT) and provides a probabilistic framework for assessing item‐level measurement properties.

The sample size was determined according to methodological recommendations for psychometric validation studies. For confirmatory factor analysis (CFA), a minimum of 200 participants is generally recommended to obtain stable parameter estimates and reliable goodness‐of‐fit indices. For Rasch modeling and other IRT approaches, samples between 200 and 300 participants are considered adequate to ensure stable item calibration, precise estimation of item difficulty, and reliable fit statistics. Therefore, the final sample of 305 participants was considered sufficient for both CFA and Rasch analyses.

All analyses were conducted using R software (version 4.3.2; R Foundation for Statistical Computing, Vienna, Austria). The following R packages were utilized:

- **eRm (v1.0‐3):** Rasch calibration and fit statistics
- **TAM (v4.3‐25):** Rasch modeling, person–item mapping, and supplementary assessment of local item independence using Yen's Q3 residual correlations
- **WrightMap (v1.5.0):** Visualization of person–item distributions
- **difR (v5.1‐1):** Differential Item Functioning (DIF) analysis
- **tidyverse (v2.0.0):** Data handling and visualization.

Rasch analysis was based on data from 305 women with BC. Reliability was assessed for both items and persons using separation reliability indices. Model–data fit was evaluated through mean square statistics (MNSQ infit and outfit) and standardized Z values (ZSTD infit and outfit). Acceptable MNSQ values ranged from 0.7 to 1.3, and ZSTD values within ±2.0 indicated adequate fit. Reliability was categorized as good (≥ 0.80), acceptable (0.60–0.79), or poor (< 0.60). Items with poor fit indices were identified, reviewed, and removed in a stepwise manner, after which the Rasch model was re‐fitted until satisfactory measurement properties were achieved.

Differential item functioning (DIF) analyses were performed to detect potential bias across subgroups (age, type of surgery, affected side, education, and employment). A DIF contrast greater than 0.5 logits was considered practically meaningful.

In Rasch measurement theory, two key assumptions were evaluated: unidimensionality and local item independence. Unidimensionality was assessed throughout Rasch calibration using principal component analysis (PCA) of Rasch residuals. An eigenvalue of the first residual contrast below 2.0 was considered indicative of sufficient unidimensionality.

After completion of the Rasch refinement process and establishment of the final 55‐item instrument (32 functioning items and 23 function‐affecting factor items), local item independence was formally evaluated using Yen's Q3 and adjusted Q3 residual correlation statistics. Adjusted Q3 values greater than 0.30 were considered indicative of potential local dependence. Item pairs exceeding this threshold were subsequently reviewed from both psychometric and conceptual perspectives to determine whether the observed residual correlations reflected clinically meaningful relationships between related ICF‐based constructs or undesirable item redundancy. Items were retained when elevated residual correlations were judged to reflect conceptually distinct but clinically related constructs rather than measurement redundancy.

CFA was performed using the lavaan package in R (version 4.6.1). Because the Rasch analysis was conducted on dichotomized item responses (0 = scores 0–5, 1 = scores 6–10), the CFA was performed using the same dichotomized responses. Given the binary nature of the observed variables, the Weighted Least Squares Mean and Variance adjusted (WLSMV) estimator was used, with all questionnaire items specified as ordered categorical variables, consistent with current recommendations for CFA of categorical data [41, 42, 43]. Because the questionnaire was intentionally developed as two conceptually distinct sections (functioning and function‐affecting factors), CFA was performed separately for each section to evaluate the hypothesized factor structure and structural validity of each conceptual component of the instrument. The six Rasch‐refined subscales were retained according to the theoretical framework underlying instrument development and were evaluated within their respective conceptual sections rather than being combined into a single CFA model.

Because WLSMV estimation was used, robust fit statistics were calculated for each model. Model fit was evaluated separately for each CFA model using multiple complementary fit indices, including indices of absolute, incremental, and residual‐based fit, in accordance with contemporary SEM reporting recommendations [41, 42, 43, 44] The following indices were examined:

- Chi‐square (χ^2^) and relative chi‐square (χ^2^/df), with values < 3 indicating good fit and < 5 acceptable fit [42]
- Comparative Fit Index (CFI), with values ≥ 0.90 indicating acceptable fit and ≥ 0.95 good fit [44]
- Tucker–Lewis Index (TLI; equivalent to NNFI), with the same cut‐offs as CFI [44]
- Root mean square error of approximation (RMSEA), with values < 0.05 indicating close fit, 0.05–0.08 acceptable fit, 0.08–0.10 marginal fit, and > 0.10 poor fit; 90% confidence intervals (CI) were also reported [41]
- Standardized root mean square residual (SRMR), with values ≤ 0.08 indicating acceptable fit [44].

Prior to analysis, the dataset was screened for completeness and data quality. The proportion of missing responses was negligible (< 5% across items), and no systematic missing pattern was detected. Given the very low level of missingness, analyses were conducted using complete‐case data without imputation, consistent with methodological recommendations for Rasch and CFA analyses when missing data are minimal.

Internal consistency was assessed using McDonald's Omega (ω) for each subscale and total score. Omega was calculated based on standardized factor loadings obtained from principal axis factoring, using the classical composite reliability formula incorporating factor loadings and error variances [45, 46]. Compared to Cronbach's alpha, Omega does not assume tau‐equivalence and provides a more accurate estimate of reliability in multidimensional instruments [47]. Reliability interpretation followed commonly accepted thresholds: ω ≥ 0.80 (good), 0.60–0.79 (acceptable), and < 0.60 (poor) [48]. Table 4 presents ω values alongside Rasch‐based person and item separation reliability indices.

## 3 Results

During the instrument development stage, a pool of 102 items related to the studied construct was created. Of these items, 45 pertained to functioning factors, while 57 were related to function‐affecting factors. Items were carefully reviewed in three expert panel sessions to remove duplicates, merge overlapping items, and revise ambiguous wording. These revisions followed the predefined amendment framework described in the Methods section, and the specific item‐level changes are detailed in Supporting Information S1.

The initial version of the instrument was finalized with 66 items, comprising 36 items in five dimensions for the functioning section and 30 items in two dimensions for function‐affecting factors (Supporting Information S1).

Qualitative content validity was subsequently evaluated through expert review and patient interviews. Based on expert feedback, several items were revised for clarity and conceptual precision. In some cases, multi‐concept items were divided into separate items to improve unidimensionality and measurement accuracy.

These modifications resulted in a provisional 70‐item instrument, consisting of 40 functioning items and 30 function‐affecting items.

### 3.1 Content Validity (Including Face Validity)

In line with contemporary measurement theory and COSMIN recommendations, face validity was evaluated as part of qualitative content validity, focusing on item clarity and comprehensibility from the perspective of the target population [40, 49]. Due to COVID‐19 restrictions, telephone interviews were conducted with 10 women diagnosed with breast cancer. Participants were asked to comment on item wording, clarity, interpretability, and alignment with the intended dimensions. Minor linguistic and wording refinements were implemented based on their feedback. No items were removed at this stage, as all were considered conceptually appropriate.

Content validity was further examined quantitatively using the CVR and the CVI. A total of 14 experts in breast cancer treatment and rehabilitation were invited to evaluate the items, of whom 10 completed the assessment. For CVR analysis, a minimum acceptable value of 0.62 was established based on Lawshe's critical values for a panel of 10 experts. Of the 66 items assessed, six items initially scored below this threshold. Following expert review, items 9 and 10 were revised due to their conceptual relevance, while items 36, 35, 22, and 17 from the functioning section and item 63 from the function‐affecting factors section were removed. The remaining 61 items were subsequently evaluated using the CVI and the modified Kappa coefficient. Item‐level CVI (I‐CVI) values exceeded 0.80 for all retained items, indicating high relevance, clarity, and simplicity. The scale‐level indices were S‐CVI/UA = 0.74 (range: 0.67–0.98) and S‐CVI/Ave = 0.97 (range: 0.94–0.98), reflecting strong overall content validity (Supporting Information S1).

In addition, qualitative feedback from experts led to further refinement, including merging overlapping items, clarifying ambiguous wording, and splitting multi‐concept items. These modifications resulted in a provisional 70‐item instrument (40 functioning items and 30 function‐affecting items), which was subsequently subjected to structural validity and reliability testing. The increase from 66 to 70 items occurred because several multi‐concept items were split into separate items following qualitative expert review to ensure conceptual clarity and unidimensional measurement. Not all items were retained following psychometric evaluation, as detailed below.

### 3.2 Determination of Structural Validity and Reliability

A total of 305 women diagnosed with breast cancer participated in this study. Participants' ages ranged from 20 to over 60 years, with the majority aged 41–60 years. Most participants were married (77%) and had a high school diploma or lower (82%). Employment status, type of surgery (mastectomy vs. non‐mastectomy), and the affected side of the breast were also recorded. Detailed demographic and clinical characteristics are presented in Table 1.

**Table 1:** Demographic information of the participants in the validity and reliability section of the studied tool (*n* = 305).

Variables		No	%
Age (year)	20–40	48	16
	41–60	213	70
	+60	44	14
Marital status	Single	23	8
	Married	235	77
	Others	47	15
Education	Diploma and lower	250	82
	Associate Degree and higher	55	18
Employment	Employed	49	16.1
	Housewife	256	83.9
Type of surgery	Mastectomy	168	55.1
	Non‐Mastectomy	137	44.9
Affected side	Right	147	48.2
	Left	158	51.8
Duration after primary medical treatments	6 months	18	5.9
	1–3 years	132	43.3
	4–10 years	155	50.8

The provisional questionnaire consisted of 70 items, including 40 items assessing functioning and 30 items assessing function‐affecting factors. All items were initially administered using an 11‐point numeric rating scale (0–10).

Before response‐category refinement, Item 36 from the social participation subscale demonstrated substantial misfit with the Rasch model and was therefore excluded from further analyses. This item showed inconsistent response patterns across participants and did not adequately represent the intended latent construct. Consequently, all subsequent Rasch analyses were performed using the remaining items.

Preliminary Rasch analyses using polytomous models (Partial Credit Model and Rating Scale Model) revealed substantial threshold disordering, poor category utilization, and non‐monotonic category probability curves across multiple items. Several intermediate response categories were rarely endorsed, and participants had difficulty consistently distinguishing adjacent categories. As a result, ordered thresholds could not be achieved for most items. Following systematic evaluation of different category‐collapsing strategies, a dichotomous scoring structure (0–5 = no/mild dysfunction; 6–10 = moderate/severe dysfunction) provided the most stable and interpretable solution. This approach resolved threshold disordering, improved item fit statistics, and ensured compliance with Rasch model assumptions. Therefore, all subsequent Rasch analyses were conducted using the dichotomized response structure.

In accordance with the study design, Rasch analysis was performed separately for each of the six theoretically defined subscales, including four functioning subscales (physical function, movement, activities of daily living, and social participation) and two function‐affecting subscales (environmental–personal and personal–emotional factors). This approach enabled the stepwise evaluation of item fit, threshold functioning, and measurement precision within each unidimensional construct.

To examine the assumption of unidimensionality, PCA of Rasch residuals was performed for each retained subscale. The unexplained variance of the first contrast was below the recommended cut‐off of 2.0 eigenvalue units across all subscales, supporting the unidimensionality of the Rasch‐calibrated constructs.

During the Rasch refinement process, item fit was evaluated iteratively. In the functioning section, item 15 in the movement domain and item 23 in the activities of daily living domain demonstrated misfit during the first stage of analysis. In the subsequent stage, item 27 was also identified as misfitting. These items were removed sequentially following expert consultation, resulting in a refined functioning item pool with satisfactory Rasch model fit (Table 2).

**Table 2:** Fit of the functioning section of the questionnaire based on a Rasch analysis (*n* = 305).

	Physical function	Brief descriptor	Fit of the 35‐item version		Fit of the 33‐item version		Fit of the 32‐item version		Decision
			Outfit MNSQ	Infit MNSQ	Outfit MNSQ	Infit MNSQ	Outfit MNSQ	Infit MNSQ	
1	Q1	Pain at rest	1.03	1.03					
2	Q2	Pain during activity	1.08	1.05					
3	Q3	Numbness at rest	0.94	0.97					
4	Q4	Numbness during activity	0.87	0.90					
5	Q5	Tightness/heaviness during activity	0.92	0.99					
6	Q6	Tingling sensation	0.99	0.99					
7	Q7	Swelling/edema	1.07	1.05					
8	Q8	Physical fatigue	0.87	0.98					
9	Q9	Skin/nail problems	1.02	1.01					
10	Q10	Shoulder mobility	1.01	1.01					
11	Q11	Respiratory problems	0.99	0.98					
12	Q12	Heart problems	1.15	1.08					
	Movement								
13	Q13	Overhead movements	1.08	1.06	1.15	1.11			
14	Q14	Repetitive arm movements	1.05	1.08	1.13	1.11			
15	Q15	Prolonged finger use	1.55	1.15					Removed due to misfit
16	Q16	Hand force	0.83	0.89	0.83	0.90			
17	Q17	Twisting lids	0.93	0.97	0.98	0.98			
18	Q18	Squeezing/wringing	0.70	0.83	0.70	0.84			
19	Q19	Lifting heavy objects	0.81	0.98	0.96	1.02			
	Activities of daily living								
20	Q20	Dressing	0.89	0.92	0.88	0.91	0.89	0.92	
21	Q21	Personal hygiene	0.81	0.86	0.86	0.88	0.87	0.88	
22	Q22	Household chores	0.88	0.97	1.04	1.00	1.24	1.01	
23	Q23	Outdoor tasks	1.31	1.19					Removed due to misfit
24	Q24	Cooking	0.84	0.92	0.86	0.93	0.90	0.94	
25	Q25	Caregiving	0.97	1.00	1.09	1.07	1.21	1.09	
26	Q26	Sleeping on the affected side	1.13	1.06	1.21	1.10	1.30	1.11	
27	Q27	Driving	1.13	1.08	1.37	1.14			Removed due to misfit
	**Social life and interaction/communication**								
28	Q28	Social gatherings	0.75	0.87					
29	Q29	Spousal relationship	1.07	1.07					
30	Q30	Family communication	0.60	0.86					
31	Q31	Social communication	0.60	0.79					
32	Q32	Life roles	1.06	0.97					
33	Q33	Leaving home	1.17	1.10					
34	Q34	Public transport	1.07	1.07					
35	Q35	Job‐related tasks	1.18	1.13					

*Note:* Items Q15, Q23, and Q27 were excluded stepwise due to misfit statistics exceeding recommended thresholds (MNSQ > 1.3).

*The Brief Descriptor column provides a concise summary of item content, not the full wording of questionnaire items.

Similarly, in the function‐affecting factors section, items 52, 62, and 69 demonstrated misfit during the initial stage and were subsequently removed together with items 59, 63, 61, and 60 during later iterations. This process resulted in a final set of 23 items demonstrating satisfactory Rasch model fit and measurement properties (Table 3). No evidence of DIF was observed across age, surgery type, affected side, education level, or employment status (Table 3).

**Table 3:** Fit of the section on function‐affecting factors of the questionnaire based on a Rasch analysis (*n* = 305).

			Fit of the 30‐item version		Fit of the 27‐item version		Fit of the 25‐item version		Fit of the 24‐item version		Fit of the 23‐item version		Decision
	Environmental‐personal factors	Brief descriptor	Outfit MNSQ	Infit MNSQ	Outfit MNSQ	Infit MNSQ	Outfit MNSQ	Infit MNSQ	Outfit MNSQ	Infit MNSQ	Outfit MNSQ	Infit MNSQ	
41	Q41	Appearance changes	1.02	1.01	1.01	1.01							
42	Q42	Disease/treatment duration	0.97	0.99	0.98	1.00							
43	Q43	Lack of emotional support	0.85	0.93	0.85	0.92							
44	Q44	Lack of help in daily tasks	0.99	0.97	0.99	0.96							
45	Q45	Pitying behaviors	1.02	1.02	1.02	1.02							
46	Q46	High treatment costs	0.86	0.94	0.88	0.95							
47	Q47	Limited medicine access	1.01	0.98	1.02	0.99							
48	Q48	Insurance issues	1.11	1.07	1.14	1.08							
49	Q49	Misjudgment by others	0.89	0.92	0.90	0.93							
50	Q50	Financial limits for devices	0.96	0.98	0.96	0.99							
51	Q51	Insufficient information	1.24	1.09	1.26	1.09							
52	Q52	Employer support	1.04	1.03									Removed due to misfit
53	Q53	Limited education programs	1.06	1.06	1.07	1.06							
	Personal‐emotional factors												
54	Q54	Loss of attractiveness	1.02	1.06	1.04	1.09	1.09	1.11	1.14	1.13	1.18	1.15	
55	Q55	Body image	1.08	1.08	1.11	1.11	1.12	1.13	1.14	1.14	1.20	1.18	
56	Q56	Distress/sadness	0.70	0.86	0.71	0.87	0.68	0.86	0.69	0.87	0.68	0.88	
57	Q57	Loneliness	0.91	0.97	0.95	1.00	1.00	1.02	1.03	1.02	1.03	1.05	
58	Q58	Low self‐confidence	0.85	0.88	0.84	0.91	0.86	0.93	0.89	0.94	0.90	0.95	
59	Q59	Fear of recurrence	1.30	1.12	1.39	1.17							Removed due to misfit
60	Q60	Worry about personal life	0.98	1.00	1.04	1.04	1.16	1.09	1.34	1.16			Removed due to misfit
61	Q61	Worry about disease future	1.01	1.05	1.19	1.11	1.53	1.25					Removed due to misfit
62	Q62	Feeling guilty	1.97	1.23									Removed due to misfit
63	Q63	Feeling uncared for	1.14	1.06	1.32	1.12							Removed due to misfit
64	Q64	Lack of motivation	0.70	0.86	0.72	0.88	0.73	0.87	0.72	0.88	0.73	0.88	
65	Q65	Hopelessness	0.88	0.88	0.89	0.89	0.87	0.90	0.89	0.92	0.94	0.93	
66	Q66	Sense of disability	0.92	0.99	0.94	1.03	0.91	1.01	0.90	1.02	0.87	1.00	
67	Q67	Failure in responsibilities	0.91	0.99	0.94	1.02	0.93	1.02	0.96	1.05	0.99	1.06	
68	Q68	Irritability/anger	0.86	0.98	0.93	1.01	0.99	1.03	1.06	1.06	1.08	1.06	
69	Q69	Suppressed emotions	1.44	1.18									Removed due to misfit
70	Q70	Mental fatigue	0.73	0.83	0.79	0.85	0.77	0.84	0.77	0.87	0.81	0.89	

*Note:* Items Q52, Q59, Q60, Q61, Q62, Q63, and Q69 were excluded due to persistent misfit across multiple Rasch analysis stages.

*The Brief Descriptor column provides a concise summary of item content, not the full wording of questionnaire items

Overall, the Rasch refinement process resulted in a final instrument comprising 55 retained items, including 32 items assessing functioning and 23 items assessing function‐affecting factors.

### 3.3 Assessment of Local Item Independence

Following completion of Rasch‐based item refinement, local item independence was formally evaluated for the final 55‐item instrument using Yen's Q3 and adjusted Q3 residual correlation statistics. Consistent with previous Rasch measurement recommendations, adjusted Q3 values greater than 0.30 were considered indicative of potential local dependence. The mean Q3 residual correlation was −0.016, whereas the maximum adjusted Q3 value was 0.703. Item pairs exceeding the predefined threshold were further examined through conceptual review (Supporting Information S2). The highest adjusted Q3 values were observed between Q3–Q4 (adjusted Q3 = 0.703), Q52–Q53 (adjusted Q3 = 0.694), Q35–Q36 (adjusted Q3 = 0.599), Q45–Q46 (adjusted Q3 = 0.578), and Q25–Q28 (adjusted Q3 = 0.578) (Supporting Information S2).

A subsequent content‐based review demonstrated that these correlated item pairs represented clinically related but conceptually distinct constructs within the ICF framework rather than redundant items. Specifically, Q3 and Q4 assessed numbness at rest and numbness during activity, respectively; Q35 and Q36 represented different dimensions of environmental support (emotional support vs. assistance with daily tasks); Q45 and Q46 reflected distinct aspects of body‐related emotional experiences (loss of attractiveness and body image); Q52 and Q53 assessed different personal consequences of disability (sense of disability and failure in responsibilities); and Q25 and Q28 represented different aspects of social participation (social gatherings and social communication).

Therefore, although several item pairs showed adjusted Q3 values above the predefined threshold, these residual associations were interpreted as reflecting conceptual relatedness rather than problematic local item dependence or redundancy. Consequently, all identified item pairs were retained to preserve the conceptual breadth and clinical content validity of the instrument.

Throughout the Rasch refinement process, statistical criteria were considered together with expert judgment to preserve content validity and theoretical comprehensiveness. Consequently, items were removed only when psychometric misfit was accompanied by limited conceptual contribution or redundancy. The final 55‐item instrument therefore demonstrated satisfactory Rasch model fit and acceptable measurement precision, while the observed residual correlations were considered acceptable based on their conceptual distinctiveness and clinical relevance.

The Rasch analysis provided both item separation reliability and person separation reliability indices. For the functioning section, the reliability values for Rasch item separation were satisfactory for the domains of physical function (0.66), movement (0.70), activities of daily living (0.71), and social life, communication, and interactions (0.60). However, the leisure domain, which comprised four items, yielded an unacceptable item separation reliability value of 0.42. Consequently, these items were excluded from the functioning section of the questionnaire. After removing the leisure domain, the overall item separation reliability for the remaining 32 items in the functioning section was good at 0.85, confirming item stability across domains. For the function‐affecting factors section, the item separation reliability values were also good, with 0.71 for environmental/personal factors and 0.83 for emotional/personal factors. An overall value of 0.85 was obtained for all 23 items, demonstrating that the items were well targeted to the sample and could consistently discriminate among different levels of item difficulty (Table 4).

**Table 4:** McDonald's Omega (ω) and separation reliability indices for dimensions of questionnaire.

	Number of items	Persons separation reliability	Items separation reliability	ω
Subscales of function questionnaire				
Physical function	12	0.60	0.66	0.66
Movement	6	0.36	0.70	0.80
Activities of daily living	6	0.56	0.71	0.72
Social life and interaction/communication	8	0.32	0.60	0.68
Total	32	0.85	0.85	0.85
Subscales of factors affecting function questionnaire				
Environmental and personal factors	12	0.66	0.71	0.71
Personal and emotional factors	11	0.73	0.83	0.86
Total	23	0.83	0.85	0.86

With respect to person separation reliability, values for the functioning section were 0.60 for physical function, 0.36 for movement, 0.56 for activities of daily living, and 0.32 for social life, communication, and interaction. The leisure domain produced a very low value of 0.03 and was therefore excluded based on statistical evidence and expert consensus. After this exclusion, the person separation reliability for the 32‐item functioning section improved to 0.85, indicating good measurement precision. For the function‐affecting factors, person separation reliability values were 0.66 for environmental/personal factors and 0.73 for emotional/personal factors, with an overall value of 0.83 for all 23 items (Table 4). These findings confirm that the questionnaire effectively differentiates participants according to their levels of functioning difficulties and affecting factors.

Internal consistency was evaluated using McDonald's Omega (ω), calculated based on factor loadings obtained from principal axis factoring. For the functioning section, ω values were 0.66 (physical function), 0.80 (movement), 0.72 (activities of daily living), and 0.68 (social life/interaction). Although the omega for the social life/interaction subscale is slightly lower than other domains, it remains within an acceptable range for newly developed multifactorial instruments. The lower value may reflect the diverse nature of social activities captured and the variability among participants' social engagement. The leisure subscale demonstrated poor internal consistency (ω = 0.44) and was therefore removed. The total functioning section (32 items) showed good internal consistency (ω = 0.85). For the function‐affecting factors, ω values were 0.71 for environmental/personal factors and 0.86 for personal/emotional factors, with an overall value of 0.86 for the 23‐item scale (Table 4). These findings indicate acceptable to good composite reliability across retained subscales.

### 3.4 The Person‐Question Map of the Functioning Section of the Instrument

The hierarchy of difficulty among the 32 questions in the functioning section was examined and compared with the subjects' ability levels using person‐question analysis. The map displays a vector that illustrates the subjects' ability to answer the questions on the left side and the difficulty level of the questions in the questionnaire on the right side. The questions positioned at the top and bottom of the graph represent higher and lower difficulty levels, respectively. Furthermore, a few subjects exhibited lower ability levels than the easiest question, indicating the presence of a minor floor effect in the questionnaire. This finding suggests that for a small subset of participants, even the least demanding items may not adequately capture their functional limitations. Such a pattern may reflect either severe impairment levels not fully addressed by the current items or atypical response behaviors. Nevertheless, the absence of a ceiling effect and the overall balanced distribution of items across the ability continuum indicate that the instrument has sufficient sensitivity to differentiate among participants with moderate to high levels of functioning. Because Rasch calibration was performed at the subscale level, person–item maps were first generated and evaluated separately for each functioning subscale. These subscale‐specific targeting analyses confirmed adequate alignment between item difficulty and participant ability. Only after establishing satisfactory subscale‐level targeting were the items combined to produce the domain‐level Wright map presented in Figure 2, which provides a concise summary of overall targeting patterns (Figure 2).

**Figure 2:**
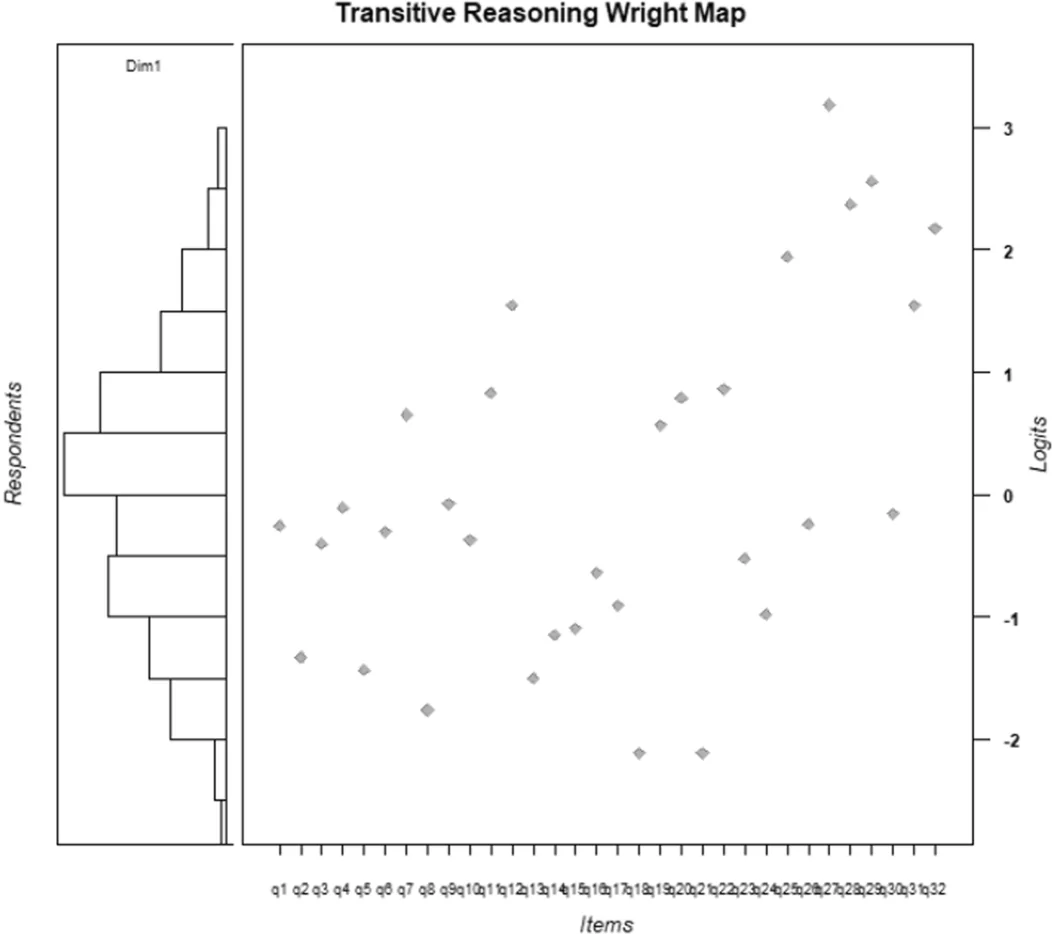
Rasch model question‐person map for the functioning section of the questionnaire.

### 3.5 The Person‐Question Map of Function‐Affecting Factors

Similarly, the hierarchy of difficulty among 23 questions related to function‐affecting factors was examined and compared with the subjects' ability levels. The figure also reveals that a small number of subjects had lower ability levels than the easiest question, again suggesting a limited floor effect. This outcome highlights that for these participants, the instrument may underestimate the influence of external or emotional factors on functioning. However, the majority of items were located at moderate difficulty levels, ensuring adequate coverage for most respondents. Targeting for the environmental/personal and emotional/personal subscales was first examined independently. These subscale‐level analyses confirmed appropriate item coverage and unidimensionality. Figure 3 therefore represents an aggregated domain‐level Wright map summarizing overall targeting after subscale calibration had been completed (Figure 3).

**Figure 3:**
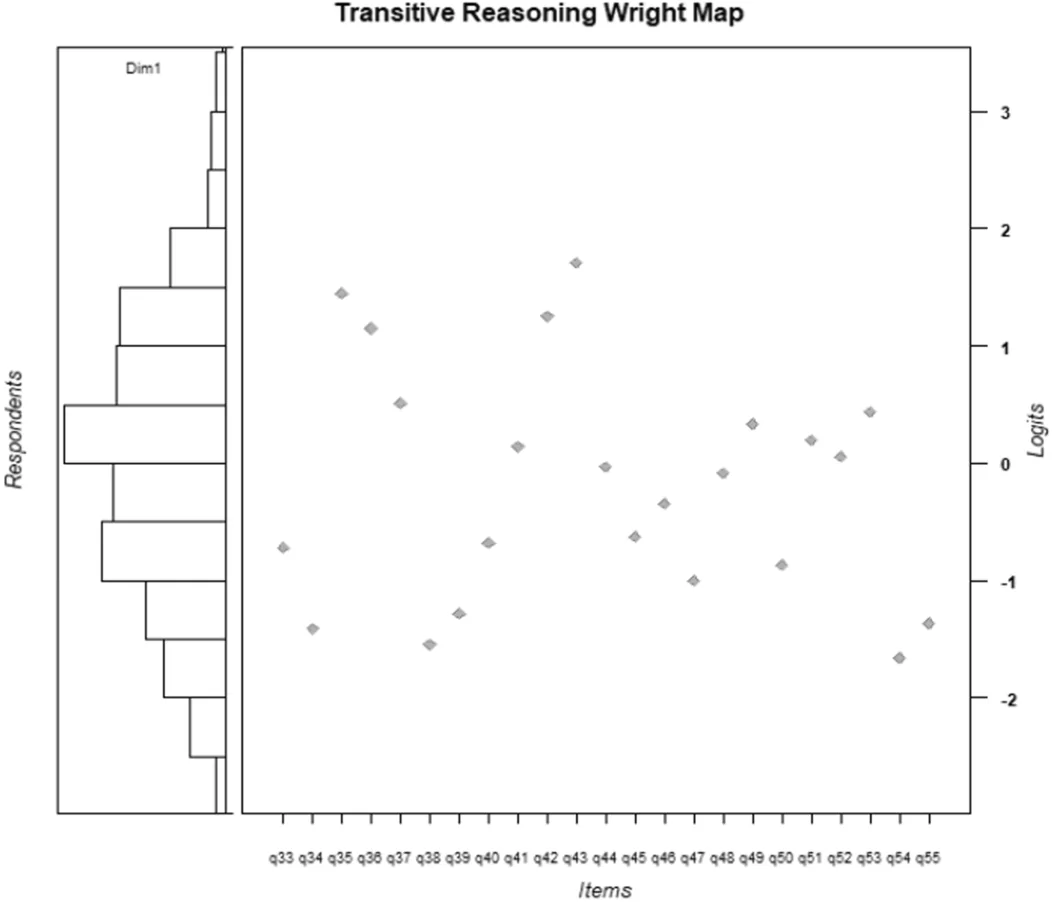
Rasch model question‐person map for the section on function‐affecting factors of the questionnaire.

It should be emphasized that all Rasch analyses including item fit, threshold ordering, unidimensionality verification, and targeting were conducted at the subscale level, consistent with the requirements of Rasch measurement theory. Since each of the six subscales represents a distinct unidimensional construct, evaluating the item set at the broader two‐domain level (functioning vs. function‐affecting factors) would violate the unidimensionality assumption. Therefore, targeting analyses and person–item distributions were first examined independently for each subscale. Subscale‐specific Wright maps and threshold assessments confirmed that items adequately targeted participant ability levels, with only minor floor effects and no ceiling effects. To avoid redundancy, only two domain‐level Wright maps (Figures 2 and 3) are presented in the manuscript as visual summaries of targeting after satisfactory subscale‐level calibration.

Floor and ceiling effects were also assessed. A small floor effect was detected, as a few respondents scored lower than the easiest item in both functioning and function‐affecting factors. No meaningful ceiling effect was observed, with only 1.3% in the functioning scale and 2.1% in the function‐affecting factors scale reaching maximum scores. These percentages were calculated based on the number of participants who achieved the minimum or maximum possible raw scores in each scale, rather than being derived directly from the Wright maps. These values are below the commonly accepted 15% threshold for problematic ceiling effects.

### 3.6 Confirmatory Factor Analysis

After completion of Rasch‐based item refinement and confirmation of local item independence, the final instrument consisted of 32 functioning items and 23 function‐affecting items. CFA was then conducted separately for the two conceptual sections of the instrument.

#### 3.6.1 Justification for Performing Two Separate CFAs

Two separate CFA were conducted because the instrument was intentionally developed as two conceptually distinct sections rather than as a single multidimensional questionnaire. The first section measures functioning (physical function, movement, activities of daily living, and social participation), whereas the second assesses factors affecting functioning (environmental–personal and personal–emotional factors). These two sections represent different latent constructs, were designed for separate interpretation, and generate independent scores. Although CFA is capable of modeling multidimensional structures within a single model, the objective of the present study was not to evaluate a higher‐order construct combining all six subscales. Instead, CFA was performed separately to confirm the hypothesized factor structure within each conceptual section of the instrument. This approach was consistent with the theoretical framework underlying the instrument, the intended interpretation of the scores, and the distinction between functioning and function‐affecting factors. It also reduced model complexity and provided more stable parameter estimation relative to the available sample size.

This structure was subsequently evaluated using CFA. CFA models were estimated using the WLSMV estimator based on the dichotomized item responses retained after Rasch analysis. Model fit was assessed using commonly recommended indices, including Comparative Fit Index (CFI), Tucker–Lewis Index (TLI), Root Mean Square Error of Approximation (RMSEA) with 90% confidence intervals (CI), and Standardized Root Mean Square Residual (SRMR) [42, 44].

For the functioning section, the modified CFA model demonstrated satisfactory overall model fit (χ^2^ = 680.32, df = 457, CFI = 0.976, TLI = 0.974, RMSEA = 0.040, 90% CI = 0.034–0.046). These findings provide preliminary support for the proposed four‐factor structure of the functioning section. Although the SRMR value (0.113) was above the conventional threshold, the excellent incremental fit indices (CFI and TLI) and low RMSEA suggested an acceptable overall representation of the hypothesized measurement model. However, interpretation of structural validity was considered together with the magnitude of individual standardized factor loadings, as some items demonstrated relatively lower contributions to their respective latent factors (Table 5; Supporting Information S3).

**Table 5:** Confirmatory factor analysis (CFA) model fit indices and standardized factor loading ranges for the functioning and function‐affecting factors sections of the questionnaire.

Questionnaire section	Factor structure	No. of items	Standardized factor loading range	χ^2^ (df)	CFI	TLI	RMSEA (90% CI)	SRMR
Functioning	Physical, movement, ADL, social	32	0.30–0.99	680.32 (457)	0.976	0.974	0.040 (0.034–0.046)	0.113
Function‐affecting factors	Environmental‐personal, Personal‐emotional	23	0.27–0.85	429.14 (226)	0.979	0.977	0.054 (0.046–0.062)	0.103

Standardized factor loadings ranged from 0.30 to 0.99 across the four latent dimensions (Supplementary 3). Although most items demonstrated moderate to high standardized factor loadings, three items (Q12, Q26, and Q32) showed relatively low loadings (0.30–0.39). These relatively low loadings indicate weaker associations between these items and their intended latent factors. Nevertheless, these items were retained because they demonstrated acceptable Rasch fit statistics, satisfactory content validity, and represented clinically important aspects of functioning that were considered essential for preserving content coverage during this initial stage of instrument development.

For the function‐affecting factors section, the revised CFA also demonstrated acceptable model fit (χ^2^ = 429.14, df = 226, CFI = 0.979, TLI = 0.977, RMSEA = 0.054, 90% CI = 0.046–0.062). Although the SRMR value (0.103) remained slightly above the recommended cut‐off, the excellent CFI and TLI values together with the acceptable RMSEA provide preliminary support for the proposed two‐factor structure (Table 5; Supporting Information S4).

Standardized factor loadings ranged from 0.27 to 0.90 across the two latent dimensions (Supporting Information S4). Similarly, several environmental‐personal items (Q40, Q43, and Q44) demonstrated relatively low standardized loadings. These findings suggest weaker associations with the underlying latent construct; however, these items address conceptually important environmental barriers identified during qualitative development and demonstrated satisfactory Rasch model fit. Therefore, these items were retained to preserve conceptual coverage of the ICF‐based framework and should be re‐evaluated in future validation studies using independent samples.

## 4 Discussion

The present study developed and psychometrically evaluated a two‐part instrument specifically designed to assess functioning and function‐affecting factors in women with breast cancer, based on the comprehensive ICF Core Set for Breast Cancer. Items were generated through a systematic review and qualitative interviews with women with breast cancer and rehabilitation specialists, ensuring conceptual relevance and clinical applicability [24, 35]. The psychometric evaluation provided preliminary evidence supporting the validity and reliability of the instrument. Rasch analysis demonstrated satisfactory item functioning and measurement properties, whereas CFA provided preliminary support for the hypothesized multidimensional structure following theoretically justified model refinement. Although additional validation in independent samples is warranted, the findings suggest that the instrument has potential for use in clinical practice and research; however, further validation of the factor structure is required before broader application can be recommended.

The findings of Rasch analysis highlighted the importance of refining measurement instruments through rigorous psychometric methods. Misfitting items identified during the analysis were removed, resulting in a more coherent factor structure and improved measurement precision. In line with previous Rasch‐based validation studies, the response categories were dichotomized to ensure proper functioning of the rating scale. Initial analyses revealed disordered thresholds in the polytomous response structure, suggesting that respondents had difficulty consistently differentiating between adjacent categories. Dichotomization effectively resolved these threshold disorders, enhancing scale stability and improving the accuracy of item–person estimates. Although collapsing response categories reduces the granularity of measurement, Rasch methodology supports such modifications when thresholds are disordered or categories are underutilized [50, 51, 52]. The internal consistency of the instrument, confirmed by McDonald's Omega (ω) was acceptable across all subscales, and the absence of significant DIF across demographic and clinical variables further strengthened the robustness of the instrument. Furthermore, the assessment of local item independence using Yen's Q3 and adjusted Q3 residual correlations identified several item pairs with elevated residual correlations above the predefined threshold, indicating potential local dependence. These item pairs were subsequently examined from both psychometric and conceptual perspectives. The content‐based review suggested that the observed associations reflected clinically related but conceptually distinct constructs within the ICF framework rather than clear item redundancy. Therefore, these items were retained primarily because of their conceptual and clinical relevance and their contribution to the breadth of construct representation, rather than solely on the basis of their statistical properties. Nevertheless, the presence of elevated residual correlations should be acknowledged as a potential source of local dependence, and future studies using independent samples and complementary analyses may further evaluate these item relationships and determine whether any refinement is warranted without compromising the content validity of the instrument.

When compared with existing generic tools such as DASH, WHODAS 2.0, and Quick‐DASH [27, 28, 29, 30, 31], as well as disease‐specific measures such as Lymph‐ICF [26, 33, 34] and BCSQ‐BC [25], the present instrument offers several distinct advantages. Many existing scales place greater emphasis on physical functioning and activity participation, while overlooking personal and contextual factors that significantly influence quality of life among BC survivors. In addition, some of these tools relied on qualitative rather than quantitative evaluation of content validity [25, 26], or demonstrated insufficient responsiveness in this population [32]. By contrast, our instrument not only captured physical aspects of functioning but also systematically incorporated emotional, social, and environmental dimensions that are critical to understanding the lived experiences of BC women. These findings reinforce the distinct contribution of the present instrument in addressing domains insufficiently covered by previous tools.

Another strength of the present tool lies in its grounding in patient‐centered evidence. Unlike instruments that adapt or extend existing items without adequately reflecting the perspectives of BC patients, this questionnaire was developed from the ground up through qualitative interviews and expert consultations [35]. This ensured that the instrument addressed domains that are often neglected in other measures, such as psychological well‐being and personal factors, while maintaining alignment with the ICF framework. As such, the tool provides a more holistic and comprehensive assessment of functioning, extending beyond acute physical outcomes to encompass the long‐term psychosocial challenges faced by BC survivors.

The psychometric rigor applied in this study further supports the utility of the developed instrument. The integration of Rasch analysis and CFA provided complementary sources of validity evidence. Rasch analysis supported the measurement properties of individual items, whereas CFA evaluated the proposed latent structure of the questionnaire. Following theoretically justified model refinement based on modification indices, both questionnaire sections demonstrated improved model fit, providing preliminary support for the proposed multidimensional structure. Nevertheless, CFA results should be interpreted as providing preliminary evidence of structural validity, and future studies should further examine the stability of the factor structure in independent populations [44, 53, 54]. This methodological approach goes beyond traditional psychometric testing by identifying items that do not contribute effectively to the measurement of underlying constructs, thereby improving both reliability and validity. In doing so, the tool offers a more precise evaluation of functioning and function‐affecting factors in women with breast cancer. The use of separate CFAs further supported the structural validity of the two conceptually distinct sections of the instrument, consistent with the theoretical framework on which the questionnaire was developed. This analytical strategy was selected to evaluate each conceptual component independently rather than to model a single overarching latent construct.

Despite the improved global model fit, several items continued to demonstrate relatively low standardized factor loadings. Within the functioning section, items assessing heart problems (Q12), spousal relationship (Q26), and job‐related tasks (Q32) showed comparatively weaker associations with their respective latent factors. Similarly, within the function‐affecting factors section, items related to insurance issues (Q40), insufficient information (Q43), and limited educational programs (Q44) also demonstrated relatively low factor loadings. Lower factor loadings indicate weaker associations with the underlying latent constructs; however, these items were retained because they demonstrated acceptable Rasch model fit, represented clinically meaningful concepts identified during qualitative instrument development, and preserved the conceptual breadth of the ICF Core Set for Breast Cancer. Consequently, although these items contributed less strongly to the latent constructs, their retention was considered theoretically justified during this stage of instrument development. Future validation studies using independent samples should further examine whether refinement or replacement of these items could further improve the structural validity of the questionnaire.

From a clinical perspective, this instrument has the potential to significantly enhance rehabilitation assessment and planning. The inclusion of domains related to emotional health, social participation, and environmental influences allows clinicians to capture challenges that persist long after the completion of medical treatment. These aspects are essential for understanding the full impact of breast cancer on survivors' daily lives, as psychosocial issues and barriers to social reintegration remain among the most significant unmet needs [35]. By providing standardized, ICF‐based data, the tool also facilitates communication within multidisciplinary teams and supports evidence‐based rehabilitation interventions.

In summary, the present study contributes to the existing body of literature by addressing a critical gap in the assessment of functioning among women with BC. While prior instruments provided partial insights, this study offers a comprehensive, psychometrically validated tool that integrates physical, emotional, and contextual factors. The findings suggest that the instrument has the potential to be useful in clinical and research settings; however, additional validation in independent populations is required before broader application can be recommended.

### 4.1 Strengths and Limitations of the Study

To ensure that the instrument assessed stable survivorship‐related functioning rather than transient treatment effects, this study included only women who had completed active treatment at least 6 months prior to participation. Consequently, the findings may not be directly generalizable to patients undergoing active treatment, whose functional status can be strongly influenced by acute treatment‐related symptoms. Although local item independence was formally evaluated using Yen's Q3 and adjusted Q3 residual correlation statistics, several item pairs demonstrated elevated residual correlations. Conceptual review indicated that these relationships reflected clinically meaningful overlap between related but distinct ICF‐based concepts rather than redundant items; therefore, the items were retained to preserve content coverage. Nevertheless, future studies using independent samples and complementary qualitative approaches may further examine whether these related concepts can be optimized without compromising construct representation.

Although CFA demonstrated improved model fit after incorporating theoretically justified residual covariances identified through inspection of modification indices, these post hoc model modifications require replication in independent samples before they can be considered stable. Therefore, the structural validity findings should be regarded as preliminary until further validation is conducted in external populations.

Although qualitative face validity was assessed through interviews with women with breast cancer to evaluate item clarity and comprehensibility, the quantitative content validation process (CVR and CVI) relied primarily on expert panel ratings. Consequently, the questionnaire may not fully capture variations in how individuals with different levels of health literacy, cultural backgrounds, or lived experiences interpret specific items. Future studies should further examine item comprehensibility and interpretability in more diverse patient populations and across different sociocultural settings to strengthen the instrument's applicability

A major strength of the present study was the integration of patient perspectives with the expertise of rehabilitation professionals during instrument development. By incorporating the experiences of women with breast cancer together with the opinions of clinicians and rehabilitation specialists, the questionnaire achieved broader conceptual coverage of functioning and function‐affecting factors based on the ICF Core Set for Breast Cancer. This approach enhanced the clinical relevance of the instrument and increased its potential usefulness for rehabilitation assessment and intervention planning.

Future studies using new and independent samples are recommended to further examine the stability and generalizability of the proposed factor structure using CFA. Replication of the CFA models in independent populations will help determine whether the theoretically justified residual covariance modifications identified in the present study remain stable across different samples. In addition, several items with relatively low standardized factor loadings (e.g., Q12, Q32, Q40, and Q43) should be re‐examined in future validation studies to determine whether wording refinement, alternative item formulations, or replacement items could further improve structural validity while preserving the conceptual coverage of the ICF domains.

It is noteworthy that this instrument was designed and psychometrically evaluated specifically for women with breast cancer who are in the chronic stage of the disease and have a stable condition. Therefore, the development of an additional instrument may be required to assess the functional conditions and factors affecting function in women with breast cancer who are undergoing active treatment processes such as surgery, radiotherapy, and chemotherapy. As this study was conducted solely in a metropolis of Iran, a similar country‐wide study is necessary to generalize the findings to the broader context of Iran and other countries.

### 4.2 Implications

This instrument has been designed and psychometrically assessed based on the ICF core set for BC. Utilizing this tool enables therapists, particularly in rehabilitation, to understand how to effectively implement bottom‐up approaches (items that assess or treat body functions and structures) or top‐down approaches (items that evaluate or address activities, participation, and environmental factors) in their treatment processes. Furthermore, the common language of the ICF, which is widely recognized in the medical field, can facilitate communication among individual specialists within the rehabilitation team, allowing them to share the results of their assessments and interventions.

## 5 Conclusion

This study demonstrates the practicality of the Rasch measurement model in assessing the scale's validity and reliability concerning functioning and function‐affecting factors. The goodness of fit indicators suggest that the construct components of the instrument meet the requirements of the Rasch measurement model. Evaluation of local item independence using Yen's Q3 statistics identified several elevated residual correlations; however, subsequent conceptual review indicated that these relationships reflected clinically related but distinct constructs, supporting retention of the items and the overall adequacy of the final Rasch model. Overall, the present findings provide preliminary evidence supporting the reliability and structural validity of the instrument for assessing functioning and function‐affecting factors in women with breast cancer. Rasch analysis demonstrated satisfactory measurement properties, and CFA provided support for the proposed multidimensional structure following theoretically justified model refinement. Nevertheless, additional validation in independent populations is warranted before broader clinical and research implementation.

## Author Contributions

All authors contributed to the concept and design of the study. The design, methodology and analysis were conducted in two parts: the development of the tool, including face and content validity, was carried out by FJ, MF, MHA, and SAH, while the assessment of construct validity and reliability was performed by EB, and FJ. Data collection was conducted by FJ with the assistance and supervision of AHE, KR, MM, FS, and ME. The first draft of the article was written by FJ, MF, and EB, and all authors provided feedback on previous versions of the article. Ultimately, all authors read and approved the final version.

## Funding

The authors have nothing to report.

## Ethics Statement

To start sampling, the necessary permissions and ethical code(IR.USWR.REC.1398.198) were obtained from the Ethics Committee of the University of Social Welfare and Rehabilitation Sciences (Tehran) for the necessary coordination.

## Consent

Informed consent was obtained from all individual participants who participated in the study.

## Conflicts of Interest

The authors declare no conflicts of interest.

## Supporting information


Supporting File 1

Supporting File 2

Supporting File 3

Supporting File 4

## Data Availability

The data that support the findings of this study are available on reasonable request from the corresponding author.
