# Supplementary material for: Development and Psychometric Assessment of a Measurement Instrument for Functioning and Function‐Affecting Factors in Women With Breast Cancer: A Rasch Analysis Approach
Source: J Eval Clin Pract. 2026 Oct 28;32(7):e70705. doi: 10.1111/jep.70705 (PMC13716315; doi:10.1111/jep.70705)
Supplement: Supplementary file 1 [file JEP-32-0-s001.docx]

| **Supplementary 4: Standardized factor loadings of the Function-affecting factors section (modified CFA model)** | | | |
| --- | --- | --- | --- |
| **Factor** | **Item** | **Standardized loading** | **P-value** |
| Environmental-personal | Q33 | 0.733 | <0.001* |
| Environmental-personal | Q34 | 0.789 | <0.001 |
| Environmental-personal | Q35 | 0.558 | <0.001 |
| Environmental-personal | Q36 | 0.410 | <0.001 |
| Environmental-personal | Q37 | 0.539 | <0.001 |
| Environmental-personal | Q38 | 0.548 | <0.001 |
| Environmental-personal | Q39 | 0.461 | <0.001 |
| Environmental-personal | Q40 | 0.312 | <0.001 |
| Environmental-personal | Q41 | 0.728 | <0.001 |
| Environmental-personal | Q42 | 0.556 | <0.001 |
| Environmental-personal | Q43 | 0.265 | 0.007 |
| Environmental-personal | Q44 | 0.333 | <0.001 |
| Personal-emotional | Q45 | 0.663 | <0.001* |
| Personal-emotional | Q46 | 0.667 | <0.001 |
| Personal-emotional | Q47 | 0.850 | <0.001 |
| Personal-emotional | Q48 | 0.770 | <0.001 |
| Personal-emotional | Q49 | 0.807 | <0.001 |
| Personal-emotional | Q50 | 0.840 | <0.001 |
| Personal-emotional | Q51 | 0.805 | <0.001 |
| Personal-emotional | Q52 | 0.647 | <0.001 |
| Personal-emotional | Q53 | 0.618 | <0.001 |
| Personal-emotional | Q54 | 0.664 | <0.001 |
| Personal-emotional | Q55 | 0.828 | <0.001 |
