# Supplementary material for: Development and Psychometric Assessment of a Measurement Instrument for Functioning and Function‐Affecting Factors in Women With Breast Cancer: A Rasch Analysis Approach
Source: J Eval Clin Pract. 2026 Oct 28;32(7):e70705. doi: 10.1111/jep.70705 (PMC13716315; doi:10.1111/jep.70705)
Supplement: Supplementary file 2 [file JEP-32-0-s002.docx]

| **Supplementary 1: Quantitative and qualitative content validity results of the preliminary instrument (66 items) assessed by 10 medical and rehabilitation specialists.** | | | | | |
| --- | --- | --- | --- | --- | --- |
|  | **Dimensions and Items** | **Modified items in qualitative content validity** | **CVR** | **I-CVI** | **Modified kappa coefficient** |
|  | **Tool: Function section** |  |  |  |  |
|  | **Physical function (hand and upper limb of the affected side)** | Edited |  |  |  |
| 1 | Pain at rest | Edited | 1 | 0.9 | 0.89 |
| 2 | Pain during daily activities | Edited | 1 | 0.9 | 0.89 |
| 3 | Numbness at rest (a sense of stretching, prickling, or heaviness) | These two items (3,4) became 3 items | 1 | 0.9 | 0.89 |
| 4 | Numbness during daily activities (a sense of stretching, prickling, or heaviness) |  | 1 | 0.9 | 0.89 |
| 5 | Tingling and throbbing | Edited | 1 | 0.9 | 0.89 |
| 6 | Swelling in the upper limb on the affected side (edema) | Edited | 0.8 | 1 | 1 |
| 7 | Fatigue in the upper limb on the affected side (reduced physical strength) | Edited | 1 | 1 | 1 |
| 8 | Skin problems (changes in skin color such as darkening, dryness, fragility, or stiffness) | Edited | 1 | 0.9 | 0.89 |
| 9 | Heart problems (increased heart rate and palpitations) | Removed at CVR stage but re-added based on experts’ opinion | 0 | 0.9 | 0.89 |
| 10 | Respiratory problems (dyspnea or severe coughing) | Removed at CVR stage but re-added based on experts’ opinion | 0 | 0.9 | 0.89 |
|  | **Movement** | Unchanged |  |  |  |
| 11 | Performing overhead movements (raising the hands above shoulder level, such as taking items from the top shelves of refrigerators or cabinets) | Unchanged, but a related item was added to physical function | 1 | 1 | 1 |
| 12 | Reaching behind the neck or back (e.g., fastening buttons or tying undergarments) | Edited | 0.8 | 1 | 1 |
| 13 | Performing prolonged finger movements (e.g., writing, drawing, chopping vegetables, grating, holding books/phones for long periods) | Unchanged | 1 | 1 | 1 |
| 14 | Pressing hard objects with the affected hand (e.g., pushing a door) | Unchanged | 1 | 0.9 | 0.89 |
| 15 | Twisting lids on stiff bottles | Unchanged | 0.8 | 1 | 1 |
| 16 | Squeezing or wringing clothes, cleaning clothes, etc. | Unchanged | 0.8 | 0.9 | 0.89 |
| 17 | Lifting and carrying heavy objects weighing more than 2 kg | Deleted | 0.6 |  |  |
| 18 | Lifting and carrying heavy equipment and loads heavier than 2 kg | Edited | 0.8 | 0.8 | 0.78 |
| 19 | Performing repetitive/prolonged activities (walking, stairs, tasks at home/work) | Removed; split into 3 items in other domains ( general /domestic duties, social life and leisure) | 1 | 1 | 1 |
|  | **General/domestic duties** | Edited |  |  |  |
| 20 | Dressing | Edited | 1 | 1 | 1 |
| 21 | Personal hygiene (e.g., bathing, showering, washing hair) and grooming/beauty care (e.g., waxing) | Edited | 1 | 0.9 | 0.89 |
| 22 | Use of the toilet | Deleted | 0.4 |  |  |
| 23 | Doing household chores (dusting, cleaning, washing dishes, making the bed, sweeping) | Unchanged | 1 | 1 | 1 |
| 24 | Cooking (e.g., moving heavy pots, sieving rice, exposure to stove heat, prolonged standing) | Edited | 0.8 | 0.9 | 0.89 |
| 25 | Caring for others (e.g., hugging babies, caring for children and others) | Unchanged | 1 | 1 | 1 |
| 26 | Sleeping on the affected side | Unchanged | 1 | 0.9 | 0.89 |
|  | **Social life and interaction/communication** | Unchanged |  |  |  |
| 27 | Attending parties | These two items (27,28) were merged and the title of the item was edited | 1 | 1 | 1 |
| 28 | Participation in religious meetings and ceremonies |  | 0.8 | 1 | 1 |
| 29 | Spousal relationships (including intimacy and sexual relations) | Converted into two items | 1 | 0.9 | 0.89 |
| 30 | Communication with family members (e.g., parents, siblings, children) | Unchanged | 1 | 1 | 1 |
| 31 | Communication with others (friends, relatives, acquaintances) | Unchanged | 1 | 1 | 1 |
| 32 | Communication with colleagues | It was converted into two items and their titles were edited | 1 | 1 | 1 |
|  | **Leisure** | Unchanged |  |  |  |
| 33 | Sports activities (e.g., yoga, aerobics, swimming, badminton, or other vigorous sports) | Converted into two items with edited titles | 1 | 0.9 | 0.89 |
| 34 | Finger-related leisure activities (e.g., sewing, knitting) | Edited | 1 | 1 | 1 |
| 35 | Studying, reading newspapers, magazines and solving tables | Deleted | 0.6 |  |  |
| 36 | Listening to music, playing musical instruments | Deleted | 0.6 |  |  |
|  | **Tool: the** **section on function-affecting factors** |  |  |  |  |
|  | **Environmental and personal factors** | Unchanged |  |  |  |
| 37 | Changes in appearance (e.g., breast removal or swelling of the affected side) | Edited | 1 | 1 | 1 |
| 38 | The long duration of the disease and treatment process | Unchanged | 1 | 1 | 1 |
| 39 | Lack of emotional support from spouse, children, or family | Unchanged | 1 | 1 | 1 |
| 40 | Lack of help and support from spouse, children, or family in daily activities | Unchanged | 1 | 1 | 1 |
| 41 | Compassionate and sympathetic behaviors of companions | Unchanged | 1 | 1 | 1 |
| 42 | High costs of disease treatment | Unchanged | 1 | 1 | 1 |
| 43 | Limited access to medicines from pharmacies | Unchanged | 1 | 1 | 1 |
| 44 | Lack of coverage of treatment costs by health insurance providers | Unchanged | 1 | 1 | 1 |
| 45 | Incorrect judgments of companions about the disease | Unchanged | 1 | 1 | 1 |
| 46 | Financial inability to provide assistive devices (e.g., prostheses, bandages, compression sleeves) | Unchanged | 1 | 1 | 1 |
| 47 | Insufficient information about the disease and its treatments from healthcare providers | Unchanged | 1 | 1 | 1 |
| 48 | Lack of support from employers regarding patient’s condition | Unchanged | 1 | 1 | 1 |
| 49 | Limited access to disease-related educational programs (e.g., group therapy) | Unchanged | 1 | 1 | 1 |
|  | **Personal-emotional factors** | Unchanged |  |  |  |
| 50 | Sense of losing physical attractiveness (e.g., distorted appearance, ill-fitting clothing) | Unchanged | 1 | 1 | 1 |
| 51 | Sense of being incomplete | Edited | 1 | 1 | 1 |
| 52 | Feelings of distress and sadness | Unchanged | 1 | 1 | 1 |
| 53 | Sense of loneliness | Unchanged | 1 | 1 | 1 |
| 54 | Decreased self-confidence | Unchanged | 1 | 1 | 1 |
| 55 | Feelings of fear or panic | Edited | 0.8 | 1 | 1 |
| 56 | Feeling worried | Edited, split into 2 items | 1 | 1 | 1 |
| 57 | Feeling guilty | Unchanged | 0.8 | 0.9 | 0.89 |
| 58 | Feeling like a burden to the family | Edited | 0.8 | 1 | 1 |
| 59 | Lack of motivation and impatience | Unchanged | 1 | 1 | 1 |
| 60 | Sense of hopelessness | Edited | 1 | 1 | 1 |
| 61 | Sense of disability and disabled | Unchanged | 1 | 1 | 1 |
| 62 | Feeling defeated | Edited | 0.8 | 1 | 1 |
| 63 | Feeling ashamed in front of spouse and family | Deleted | 0.6 |  |  |
| 64 | Irritability, anger, or loss of temper | Unchanged | 1 | 1 | 1 |
| 65 | Non-expression of emotions (not discussing personal feelings or daily life events) | Unchanged | 1 | 1 | 1 |
| 66 | Fatigue (reduced physical strength) | Edited | 1 | 1 | 1 |
