# Supplementary material for: Development and Psychometric Assessment of a Measurement Instrument for Functioning and Function‐Affecting Factors in Women With Breast Cancer: A Rasch Analysis Approach
Source: J Eval Clin Pract. 2026 Oct 28;32(7):e70705. doi: 10.1111/jep.70705 (PMC13716315; doi:10.1111/jep.70705)
Supplement: Supplementary file 3 [file JEP-32-0-s003.docx]

| **Supplementary 3: Standardized factor loadings of the Functioning section (modified CFA model)** | | | |
| --- | --- | --- | --- |
| **Factor** | **Item** | **Standardized loading** | **P-value** |
| Physical | Q1 | 0.425 | <0.001 |
| Physical | Q2 | 0.441 | <0.001 |
| Physical | Q3 | 0.445 | <0.001 |
| Physical | Q4 | 0.532 | <0.001 |
| Physical | Q5 | 0.433 | <0.001 |
| Physical | Q6 | 0.539 | <0.001 |
| Physical | Q7 | 0.474 | <0.001 |
| Physical | Q8 | 0.616 | <0.001 |
| Physical | Q9 | 0.434 | <0.001 |
| Physical | Q10 | 0.620 | <0.001 |
| Physical | Q11 | 0.443 | <0.001 |
| Physical | Q12 | 0.351 | <0.001 |
| Movement | Q13 | 0.777 | <0.001 |
| Movement | Q14 | 0.789 | <0.001 |
| Movement | Q15 | 0.830 | <0.001 |
| Movement | Q16 | 0.796 | <0.001 |
| Movement | Q17 | 0.895 | <0.001 |
| Movement | Q18 | 0.785 | <0.001 |
| ADL | Q19 | 0.786 | <0.001 |
| ADL | Q20 | 0.852 | <0.001 |
| ADL | Q21 | 0.673 | <0.001 |
| ADL | Q22 | 0.754 | <0.001 |
| ADL | Q23 | 0.718 | <0.001 |
| ADL | Q24 | 0.551 | <0.001 |
| Social | Q25 | 0.885 | <0.001 |
| Social | Q26 | 0.387 | <0.001 |
| Social | Q27 | 0.834 | <0.001 |
| Social | Q28 | 0.993 | <0.001 |
| Social | Q29 | 0.661 | <0.001 |
| Social | Q30 | 0.516 | <0.001 |
| Social | Q31 | 0.602 | <0.001 |
| Social | Q32 | 0.301 | 0.005 |
