# Supplementary material for: Development and Psychometric Assessment of a Measurement Instrument for Functioning and Function‐Affecting Factors in Women With Breast Cancer: A Rasch Analysis Approach
Source: J Eval Clin Pract. 2026 Oct 28;32(7):e70705. doi: 10.1111/jep.70705 (PMC13716315; doi:10.1111/jep.70705)
Supplement: Supplementary file 4 [file JEP-32-0-s004.docx]

| **Supplementary 2: Item pairs showing potential local dependence based on Yen’s adjusted Q3 statistics** | | | | | |
| --- | --- | --- | --- | --- | --- |
| **Item pair** | **Domain** | **Item description** | **Adjusted Q3** | **Interpretation** | **Decision** |
| Q3–Q4 | Physical function | Numbness at rest / Numbness during activity | 0.703 | Related sensory symptoms assessed in different contexts | Retained |
| Q52–Q53 | Personal-emotional factors | Sense of disability / Failure in responsibilities | 0.694 | Related disability consequences but conceptually distinct | Retained |
| Q35–Q36 | Environmental-personal factors | Lack of emotional support / Lack of help in daily tasks | 0.599 | Different support dimensions | Retained |
| Q45–Q46 | Personal-emotional factors | Loss of attractiveness / Body image | 0.578 | Related body-related concerns with different meanings | Retained |
| Q25–Q28 | Social life and interaction/communication | Social gatherings / Social communication | 0.578 | Different social participation aspects | Retained |
